# Diagnostic Role of Cell-Free miRNAs in Identifying Placenta Accreta Spectrum during First-Trimester Screening

**DOI:** 10.3390/ijms25020871

**Published:** 2024-01-10

**Authors:** Angelika V. Timofeeva, Ivan S. Fedorov, Yuliya V. Suhova, Alla M. Tarasova, Larisa S. Ezhova, Tatyana M. Zabelina, Oksana N. Vasilchenko, Tatyana Y. Ivanets, Gennady T. Sukhikh

**Affiliations:** Kulakov National Medical Research Center of Obstetrics, Gynecology, and Perinatology, Ministry of Health of Russia, Ac. Oparina 4, 117997 Moscow, Russia; is_fedorov@oparina4.ru (I.S.F.); j_bezzubenko@oparina4.ru (Y.V.S.); am_tarasova@oparina4.ru (A.M.T.); l_ezhova@oparina4.ru (L.S.E.); romashova-1993@bk.ru (T.M.Z.); o_vasilchenko@oparina4.ru (O.N.V.); t_ivanets@oparina4.ru (T.Y.I.); g_sukhikh@oparina4.ru (G.T.S.)

**Keywords:** miRNA, NGS, real-time PCR, peripheral blood, placenta, myometrium, placenta accreta spectrum, first-trimester screening

## Abstract

Placenta accreta spectrum (PAS) is a severe complication of pregnancy associated with excessive invasion of cytotrophoblast cells at the sites of the endometrial–myometrial interface and the myometrium itself in cases of adherent (creta) and invasive (increta and percreta) forms, respectively. This leads to a high risk of massive blood loss, maternal hysterectomy, and preterm birth. Despite advancements in ultrasound protocols and found associations of alpha-fetoprotein, PAPP-A, hCG, PLGF, sFlt-1, IL-8, and IL-33 peripheral blood levels with PAS, there is a high need for an additional non-invasive test to improve the diagnostic accuracy and to select the real PAS from the suspected ones in the first-trimester screening. miRNA signatures of placental tissue, myometrium, and blood plasma from women with PAS in the third trimester of pregnancy, as well as miRNA profiles in exosomes from the blood serum of women in the first trimester with physiologically progressing pregnancy, complicated by PAS or pre-eclampsia, were obtained using deep sequencing. Two logistic regression models were constructed, both featuring statistically significant parameters related to the levels of miR-26a-5p, miR-17-5p, and miR-101-3p, quantified by real-time PCR in native blood serum. These models demonstrated 100% sensitivity in detecting PAS during the first pregnancy screening. These miRNAs were identified as specific markers for PAS, showing significant differences in their blood serum levels during the first trimester in the PAS group compared to those in physiological pregnancies, early- or late-onset pre-eclampsia groups. Furthermore, these miRNAs exhibited differential expression in the PAS placenta and/or myometrium in the third trimester and, according to data from the literature, control angiogenesis. Significant correlations were found between extracellular hsa-miR-101-3p and nuchal translucency thickness, hsa-miR-17-5p and uterine artery pulsatility index, and hsa-miR-26a-5p and hsa-miR-17-5p with PLGF. The developed test system for early non-invasive PAS diagnosis based on the blood serum level of extracellular miR-26a-5p, miR-17-5p, and miR-101-3p can serve as an auxiliary method for first-trimester screening of pregnant women, subject to validation with independent test samples.

## 1. Introduction

Placenta accreta spectrum (PAS) is a serious obstetric complication characterized by excessive invasive growth of chorionic villi into adjacent tissue structures. This term encompasses both abnormal adherence (placenta creta, where villi adhere to the myometrium) and abnormal invasion (placenta increta, where villi invade the myometrium; placenta percreta, where villi invade the full thickness of the myometrium) [1]. According to D.A. Carusi, the prevalence of PAS is reported as 1 in 1000 deliveries [2], with an increasing incidence observed over time. For instance, in a tertiary south Italian center, the incidence of PAS tripled from 0.12% to 0.31% between 1970 and 2000 [3]. Other authors report a 100-fold increase in PAS frequency since the 1950s [4,5], which is attributed to the rising rate of cesarean section procedures [6,7]. In turn, the risk of placenta previa significantly increases with the frequency of cesarean sections, representing an additional risk factor for PAS, constituting 50% [8]. This is associated with the increased tropism of trophoblast cells of the blastocyst to the altered scar tissue area, leading to myofiber disarray, inflammatory processes, and dystrophy of elastic and collagen fibers.

Among the etiological factors of PAS, besides changes in scar tissue, curettage, myomectomy, uterine anomalies, endometriosis, and endometritis are noted, all of which may lead to endometrial fibrosis and poor decidualization [8]. Several theories have been proposed to explain the origin of PAS One theory, which involves disorders in the coordinated regulation of extravillous trophoblast differentiation from progenitor cytotrophoblasts, resulting in excessive invasion into the myometrium to remodel the uterine vascular system, causing hypervascularity and vascular dysfunction [9,10,11]. This trophoblast behavior resembles cancer-like progression [11]. Single-cell transcriptome analysis of PAS and normally detached placenta tissues has revealed close communication between excessive numbers of two cytotrophoblast cell types (LAMB4+ and KRT6A+) and maternal stromal cell subtypes (ADIRF+ and DES+), supporting trophoblast cell migration and invasion, as well as interactions with vascular endothelial cells through FLT1-VEGFA and JAG1-NOTCH2 cell–cell interactions inducing abnormal blood vessels in the myometrium [12]. Another hypothesis suggests that abnormal vascularization with local hypoxia in the uterine scar area impacts decidualization, causing a defect in the regulatory properties of the decidua. This defect allows trophoblast cells to be more aggressive and penetrative at the sites of the endometrial–myometrial interface and the myometrium itself [13,14]. The main complications of PAS include massive blood loss, disseminated intravascular coagulation, hysterectomy, and preterm birth, leading to increased maternal and fetal morbidity and mortality [15]. Despite improvements in ultrasound protocols [1,16,17], the frequency of undiagnosed PAS before delivery is variable [18,19], partly due to the different ultrasound equipment used by ultrasound examinators, the subjective quality of the ultrasound sings of PAS, and the lack of clear evaluation criteria for each of the three grades of PAS. Therefore, the timely and accurate antenatal diagnosis of PAS is essential to formulate the correct patient management algorithm and plan delivery by a multidisciplinary team to reduce the frequency of postpartum complications as also claimed by Pavón-Gomez N. et al. [19]. Therefore, there is a high need for an additional non-invasive test to differentiate real PAS from ultrasound suspected ones antenatally, preferably in the first trimester of pregnancy.

Circulating biomolecules in maternal blood were examined for their potentials use in diagnosing PAS [20,21,22]. The sensitivity and specificity of maternal serum alpha-fetoprotein in the diagnosis of placenta previa complicated by PAS were only 71% and 46%, respectively [23]. According to a meta-analysis, pregnant women with PAS have a high serum PAPP-A level in the first trimester [24,25], suggesting that this biomarker can be recommended for identifying the risk group for developing PAS. Several studies have shown that, compared to a normal pregnancy, the level of β-hCG in maternal blood serum increases in the first and second trimesters of pregnancy during PAS [23,26]; however, blood serum hCG levels are also associated with miscarriage, ectopic pregnancy, and fetal abnormalities [21]. PlGF levels are significantly higher in subgroups with pathological placental invasion compared to the group with normal placental implantation, while sFlt-1 levels and the sFlt-1/PlGF ratio are lower [27,28,29]. IL-8 promotes migration and invasion of extravillous trophoblast cells during pregnancy, and its elevation in blood serum may serve as a biomarker for PAS [30]. The level of IL-33 is significantly higher in patients with PAS than in healthy pregnant women [31]. Despite the identified correlations between PAS and the levels of these circulating biomolecules in maternal blood, it is necessary to prove their specificity for PAS and their ability to distinguish it from other pregnancy complications.

Due to the epigenetic regulation of trophoblast differentiation, migration, and invasion [32,33], miRNAs, acting as master regulators of the human genome at the transcriptional and post-transcriptional levels, were analyzed in various biological samples to associate their levels with PAS [21,34,35,36]. However, miRNA markers for PAS have been identified in the third trimester of pregnancy in studies conducted to date. To individualize the management tactics of pregnant women, preparing for qualified surgical assistance at the time of delivery with the possibility of blood transfusion, it is optimal to conduct screening of women in the first trimester of pregnancy for the content of miRNA markers of PAS in blood serum. Therefore, the aim of this study was to identify extracellular miRNAs circulating in the peripheral blood of women in the first trimester of pregnancy, specific to placental and/or myometrial tissue, and to differentiate PAS from other pregnancy complications, such as pre-eclampsia.

## 2. Results

To identify circulating cell-free microRNA (miRNA) markers indicative of placental invasion during the first trimester of pregnancy, the study was conducted in four stages:

(I) Generation of miRNA expression patterns using deep sequencing in both placental and myometrial tissues within the region of pathological trophoblast invasion and beyond at the time of delivery in women with placenta accreta spectrum (PAS) conditions, including creta, increta, and percreta.
(II) Identification of placenta- and myometrium-specific miRNAs in the blood plasma of pregnant women using deep sequencing and quantitative real-time polymerase chain reaction (qRT-PCR) on the day of delivery, enabling the diagnosis of PAS.
(III) Qualitative and quantitative analysis of exosomal miRNA composition in the serum of women at 11–14 weeks of pregnancy, facilitating the diagnosis of PAS through deep sequencing with subsequent validation of the obtained data using qRT-PCR.
(IV) Quantitative analysis of exosomal miRNA markers for PAS in the native serum of women at 11–14 weeks of pregnancy with either a normal course or complications such as the onset of pre-eclampsia symptoms after 20 weeks of gestation or complications involving placental invasion. The goal is to construct a logistic regression model for the accurate diagnosis of PAS.

### 2.1. Analysis of miRNA Expression Patterns Using Deep Sequencing in Placental and Myometrial Tissues from Patients with Placental Invasion at the Time of Delivery

In the initial stage of the study, deep sequencing of small non-coding RNAs was employed to obtain and compare the expression profiles of microRNAs (miRNAs) in various regions of the placenta and myometrium. Tissues were collected during cesarean section procedures from women in the first cohort (Table 1) with diagnoses of placenta creta (seven patients), placenta increta (six patients), and placenta percreta (four patients).

**Figure 1:**
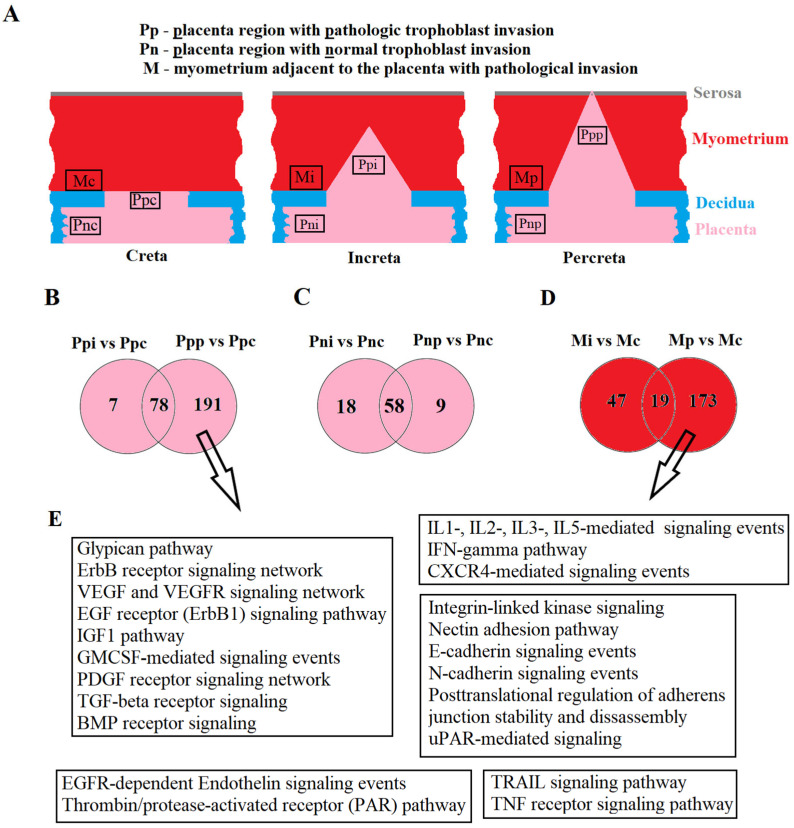
Analysis of placental and myometrial tissues on the day of delivery in women with placenta creta, increta, and percreta using deep sequencing of miRNA. Schematic representation of sample collection locations for placenta and myometrium (**A**); Venn–Euler diagrams of differentially expressed miRNAs in the placenta within the pathological trophoblast invasion site for percreta and increta relative to creta (**B**), in the placenta outside areas of pathological trophoblast invasion for percreta and increta relative to creta (**C**), in myometrial tissues adjacent to areas of pathological trophoblast invasion for percreta and increta relative to creta (**D**), common signaling pathways regulated by differentially expressed miRNAs in the pathological trophoblast invasion site for percreta (Ppp vs. Ppc) and in adjacent myometrial tissue (Mp vs. Mc), according to MirTarbase and Funrich (**E**).

Placental samples were collected from areas of pathological trophoblast invasion and from regions outside this area. Myometrial samples were obtained from areas adjacent to the placental invasion site. The schematic localization of placental and myometrial sample collection and the designation of corresponding groups are illustrated in Figure 1A.

During the analysis of miRNA expression profiles in placental tissue, two types of comparisons were performed: (1) comparison of placental areas with pathological trophoblast invasion in women with creta (seven samples), increta (six samples), and percreta (four samples): Ppp vs. Ppc, Ppi vs. Ppc; (2) comparison of placental areas outside pathological trophoblast invasion sites in women with creta (three samples), increta (four samples), and percreta (three samples): Pnp vs. Pnc, Pni vs. Pnc.

In the first type of comparison, expression profiles of miRNAs were obtained, significantly differentiating samples of Ppi from Ppc in terms of expression levels for 85 miRNAs (Table S1, Sheet 1), and Ppp from Ppc for 269 miRNAs (Table S2, Sheet 1). Among them, altered expression levels in both types of invasions (increta and percreta) were identified for 78 miRNAs (Figure 1B), constituting 92% of all differentially expressed miRNAs in the case of increta and 29% in the case of percreta. These data indicate similarities in molecular and biological changes in the placenta within the invasion area and the myometrial tissue in cases of increta and percreta, with an exacerbation of these changes in the case of percreta, involving an additional 191 miRNAs.

In the second type of comparison, expression profiles of miRNAs significantly differentiating samples of Pni from Pnc in terms of expression levels for 76 miRNAs (Table S3, Sheet 1), and Pnp from Pnc for 67 miRNAs (Table S4, Sheet 1) were obtained. Among them, altered expression levels in both types of invasions (increta and percreta) were identified for 58 miRNAs (Figure 1C), constituting 76% of all differentially expressed miRNAs in the case of increta and 87% in the case of percreta. These data suggest commonalities in molecular and biological changes throughout the entire placenta in women with increta and percreta, possibly formed during the embryo implantation stage due to the interaction of trophoblasts with a pathologically altered deciduous layer of the endometrium. Cells of the cytotrophoblast in certain areas of such a placenta acquire a phenotype with excessive invasive activity under predisposing conditions, such as structural changes in the myometrium. Therefore, deep-sequencing analysis was performed on miRNA expression levels in myometrial tissue adjacent to the site of pathological trophoblast invasion for percreta (three samples) and increta (four samples) compared to creta (three samples). Expression profiles of miRNAs significantly differentiating samples Mi from Mc (Table S5, Sheet 1) and Mp from Mc (Table S6, Sheet 1) were obtained. Among them, altered expression levels in myometrial tissue for both types of PAS (increta and percreta) were identified for only 19 miRNAs (Figure 1D), constituting 29% of all differentially expressed miRNAs in the case of increta and 10% in the case of percreta. It is noteworthy that more pronounced changes in the qualitative and quantitative composition of miRNAs in myometrial tissue adjacent to placenta percreta were observed compared to that adjacent to placenta increta, possibly contributing to a greater depth of trophoblast cell invasion in the case of placenta percreta.

For each miRNA profile in a specific type of comparison (see above), the use of the MirWalk program (http://mirwalk.umm.uni-heidelberg.de/search_mirnas/, last accessed on 15 October 2023) allowed the identification of experimentally proven target genes according to the MiRTarBase algorithm (Sheet 2 in Tables S1, S2, S5, and S6). Additionally, using the FunRich program (http://www.funrich.org/, last accessed on 15 October 2023), signaling pathways regulated by differentially expressed miRNAs in placental and myometrial tissues in different types of PAS (Sheet 3 in Tables S1, S2, S5, and S6) were revealed. Since the most significant molecular and biological changes were identified in the placenta within the pathological trophoblast invasion site for percreta and in adjacent myometrial tissue compared to other types of PAS; for clarity, in Figure 1E we have presented common signaling pathways regulated by differentially expressed miRNAs in the comparison of Ppp vs. Ppc and Mp vs. Mc. Imbalances in the activity of these signaling pathways may account for local inflammatory processes, structural rearrangements in the extracellular matrix, neovascularization, apoptosis, and changes in the proliferative and invasive properties of cells, such as cytotrophoblast cells.

### 2.2. Identification of Placenta- and Myometrium-Specific miRNAs Circulating in the Blood Plasma of Pregnant Women on the Day of Delivery Using Deep Sequencing and Real-Time Quantitative PCR

**Figure 2:**
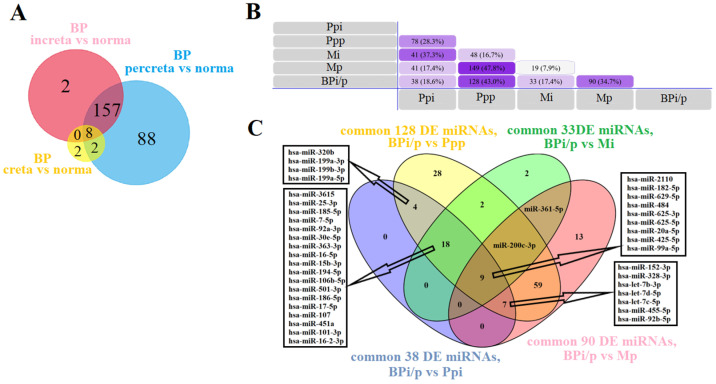
Comparison of miRNA Expression Profiles in placental tissue, adjacent myometrium, and peripheral blood plasma of Patients in Cohort 1. (**A**) Venn–Euler diagram for comparative analysis of lists of differentially expressed (DE) miRNAs in the blood plasma of women with creta, increta, and percreta relative to the group with a normal pregnancy using FunRich v. 3.1.3 (http://www.funrich.org/, last accessed on 15 October 2023) and Venny 2.1.0 (https://bioinfogp.cnb.csic.es/tools/venny/, last accessed on 15 October 2023). (**B**) Venn–Euler diagram for comparative analysis of the 165 DE miRNAs list in the blood plasma common for placenta increta and percreta cases (BPi/p, from (**A**)) with the DE miRNAs lists in placenta and myometrium for increta (Ppi and Mi, respectively) and percreta (Ppp and Mp, respectively) cases using FunRich v. 3.1.3 (http://www.funrich.org/, last accessed on 15 October 2023). (**C**) Venn–Euler diagram for comparative analysis of the DE miRNA list common to BPi/p and Ppi, BPi/p and Ppp, BPi/p and Mi, BPi/p and Mp (all obtained from (**B**)), constructed using Venny 2.1.0 (https://bioinfogp.cnb.csic.es/tools/venny/, last accessed on 15 October 2023).

With the aim of developing a non-invasive diagnostic test for PAS in the third trimester of pregnancy, expression profiles of miRNAs were obtained in the peripheral blood plasma of 24 women from the first cohort (Table 1) with various types of PAS using deep sequencing. Lists of differentially expressed (DE) miRNAs were generated for creta (Table S7, Sheet 1), increta (Table S7, Sheet 2), and percreta (Table S7, Sheet 3) relative to the group with a normal pregnancy. These lists of DE miRNAs were compared by constructing a Venn–Euler diagram (Figure 2A), indicating that most miRNAs differentially expressed in the blood plasma of women with creta and increta had significant altered expression levels in the blood plasma of women with percreta. As expression profiles of miRNAs in placental, myometrial, and blood plasma samples were analyzed in the same cohort of patients using deep sequencing, the tissue specificity of circulating blood miRNA markers for PAS was evaluated.

The common DE list of 165 miRNAs in the blood plasma (BPi/p, Figure 2A) for increta and percreta was compared with the lists of DE miRNAs in Ppi (Table S1, Sheet 1), Ppp (Table S2, Sheet 1), Mi (Table S5, Sheet 1), and Mp (Table S6, Sheet 1). Intersections were found between BPi/p and Ppi for 38 miRNAs, BPi/p and Ppp for 128 miRNAs, BPi/p and Mi for 33 miRNAs, BPi/p and Mp for 90 miRNAs (Figure 2B). For further validation of the obtained data using real-time quantitative PCR, 40 miRNAs were selected that were differentially expressed in the blood plasma of women with invasive types of PAS (increta and percreta), as well as in placenta and/or myometrium in both invasive PAS types. The list of the selected 40 miRNAs is presented in Figure 2C.

The peripheral blood plasma samples from the second cohort of patients (*n* = 46, Table 2) were used to validate the sequencing data.

**Figure 3:**
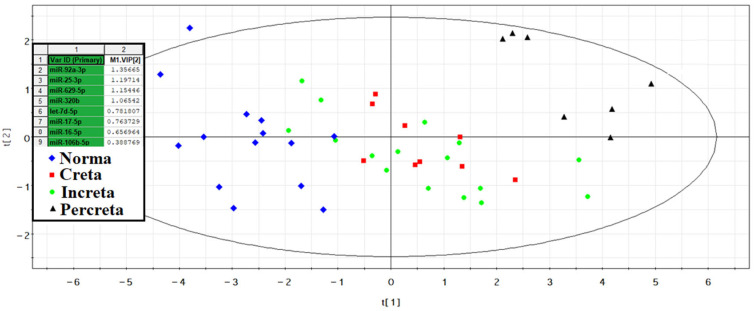
PLS-A analysis of quantitative RT-PCR data on the expression level of miRNAs in the peripheral blood plasma of pregnant women with physiological pregnancy and PAS.

The −ΔCt values were calculated based on the difference between the Ct values of the analyzed 40 miRNAs and the Ct value of the exogenous RNA UniSp6 (see Section 4) in each sample. Using the partial least squares regression (PLS-A) method, the greatest contribution to the clustering of blood plasma samples from women with different types of PAS was made by hsa-miR-92a-3p, hsa-miR-25-3p, hsa-miR-629-5p, hsa-miR-320b, hsa-let-7d-5p, hsa-miR-17-5p, hsa-miR-16-5p, hsa-miR-106b-5p (Figure 3).

**Figure 4:**
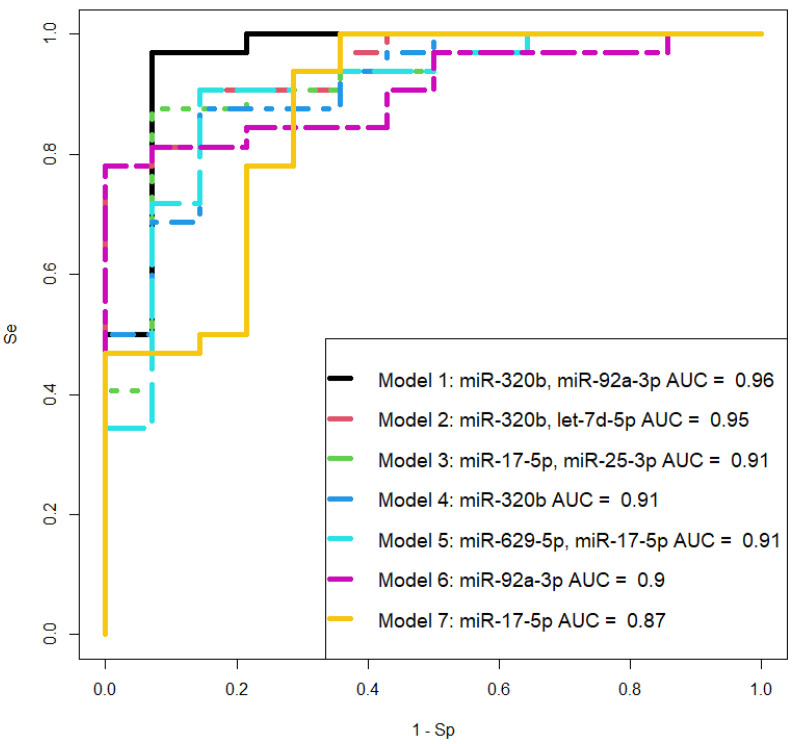
Receiver operating characteristic (ROC) curves of the logistic regression models based on real-time quantitative PCR data when comparing the combined groups “creta, increta, percreta” with the “norma” group for the content of miRNAs in the blood plasma of pregnant women in the third trimester. Se—sensitivity, Sp—specificity.

From Figure 3, it is evident that all samples of peripheral blood plasma from women with PAS differ from control samples in the expression profile of eight miRNAs, forming a distinct cluster. For this reason, it was decided to combine the creta, increta, and percreta groups to develop logistic regression models for creating a non-invasive diagnostic test for PAS based on the quantitative assessment of miRNAs in blood plasma in the third trimester of pregnancy. Using RStudio, optimal combinations of miRNAs associated with the presence of PAS in pregnant women were found step by step, considering their contribution to building logistic regression models. In these models, the dependent variable (response variable) was the presence or absence of PAS in pregnant women (0—absence of PAS; 1—presence of PAS). The selected models, presented in Figure 4, included statistically significant independent variables. The parameters of the models in Figure 4 are listed in Table 3. Formulas 1, 2, and 3, describing the models 1, 2, and 5 in Figure 4, respectively, are provided below.

(1) Model 1 formula:

(1) $$\frac{1}{1+e^{-12.43+2.6x_{1}+2.15x_{2}}}$$

wherre *x*_1_—«−∆Ct» value for hsa-miR-320b, *x*_2_—«−∆Ct» value for hsa-miR-92a-3p;

(2) Model 2 formula:

(2) $$\frac{1}{1+e^{-9.7+4x_{1}+0.69x_{2}}}$$

where *x*_1_—«−∆Ct» value for hsa-miR-320b, *x*_2_—«−∆Ct» value for hsa-let-7d-5p;

(3) Model 5 formula:

(3) $$\frac{1}{1+e^{-9.56+0.46x_{1}+0.57x_{2}}}$$

where *x*_1_—«−∆Ct» value for hsa-miR-629-5p, *x*_2_—«−∆Ct» value for hsa-miR-17-5p.

Model 1 demonstrates the highest sensitivity (96.9%) compared to Models 2 (Se = 78.1%) and 5 (Se = 78.1%), indicating a superior diagnostic value in identifying PAS in the third trimester of pregnancy. On the other hand, Models 2 and 5 exhibit 100% specificity, unlike Model 1 (Sp = 93%), ensuring 100% accuracy in detecting pregnancies without PAS. Simultaneous utilization of all three models on an extended training and testing dataset is essential for understanding their diagnostic value and selecting one for further consideration.

### 2.3. Analysis of Qualitative and Quantitative Composition of Exosome microRNA (miRNA) in the Blood Serum of Women at 11–14 Weeks of Pregnancy

One of the means of intercellular communication is the directed delivery of molecules, including miRNA, contained within exosomes. We hypothesized that morphofunctionally altered tissues of the placenta and myometrium in the area of pathological trophoblast invasion secrete exosomes with a modified miRNA profile into the blood. This profile could potentially diagnose the presence of PAS in early pregnancy stages, such as the 11–14 weeks of gestation.

A retrospective study of exosomes from 48 women’s peripheral blood serum at 11–14 weeks of pregnancy was performed, and four main groups were formed according to the diagnose at delivery (Table 4): normal pregnancy (N, 10 women); high risk of developing pre-eclampsia according to the Astraia program without clinical manifestations after 20 weeks of pregnancy (Nhr, 7 women); development of clinical symptoms of early- or late-onset pre-eclampsia after 20 weeks of pregnancy (PE, 21 women); placenta creta, increta, or percreta in pregnant women (PAS, 10 women). miRNA expression profiles were obtained using deep sequencing as described in Section 4.

**Figure 5:**
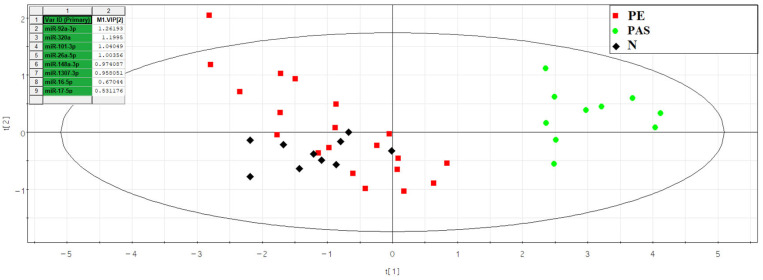
PLS analysis of deep-sequencing data of miRNAs in women’s peripheral blood serum at 11–14 weeks of pregnancy with physiological course, early- or late-onset pre-eclampsia, and PAS.

The comparison of read numbers in the “PAS” group with those in the combined “N + PE” group is presented in Table S8. The partial least squares regression (PLS) method was used to assess the contribution of each identified miRNA to the separation of samples from the analyzed groups (Figure 5).

**Figure 6:**
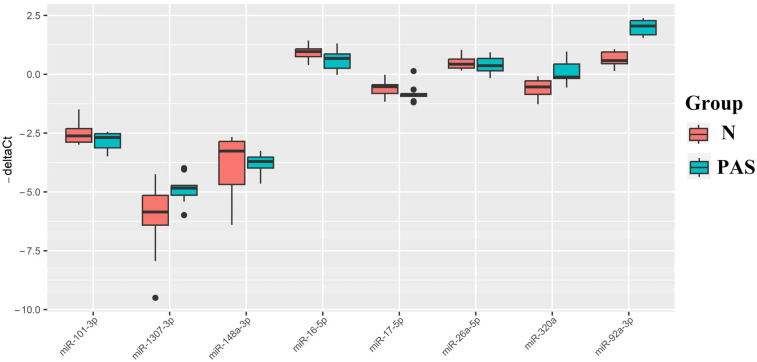
Box plot of miRNA content in exosomes of blood serum from women at 11–14 weeks of pregnancy with a physiological course and PAS. The position of the median inside the box in the form of a horizontal line and outliers in the form of points are indicated.

The distribution of samples in Figure 5 shows that blood serum samples from women with PAS are distant from all other samples and form a separate cluster. The molecules hsa-miR-92a-3p, hsa-miR-320a, hsa-miR-101-3p, hsa-miR-26a-5p, hsa-miR-148a-3p, hsa-miR-1307-3p, hsa-miR-16-5p, and hsa-miR-17-5p contributed most to this distribution. The levels of these molecules were evaluated by quantitative PCR in samples from the N and PAS groups (Figure 6). The “−ΔCt” values were calculated based on the difference between the Ct value of the analyzed miRNA and the Ct value of the endogenous hsa-let-7a-5p, given its stable expression in all analyzed samples by deep sequencing (coefficient of variation was equal to 0.165, see Table S8). The statistical significance of differences between the compared groups is presented in Table 5.

**Figure 7:**
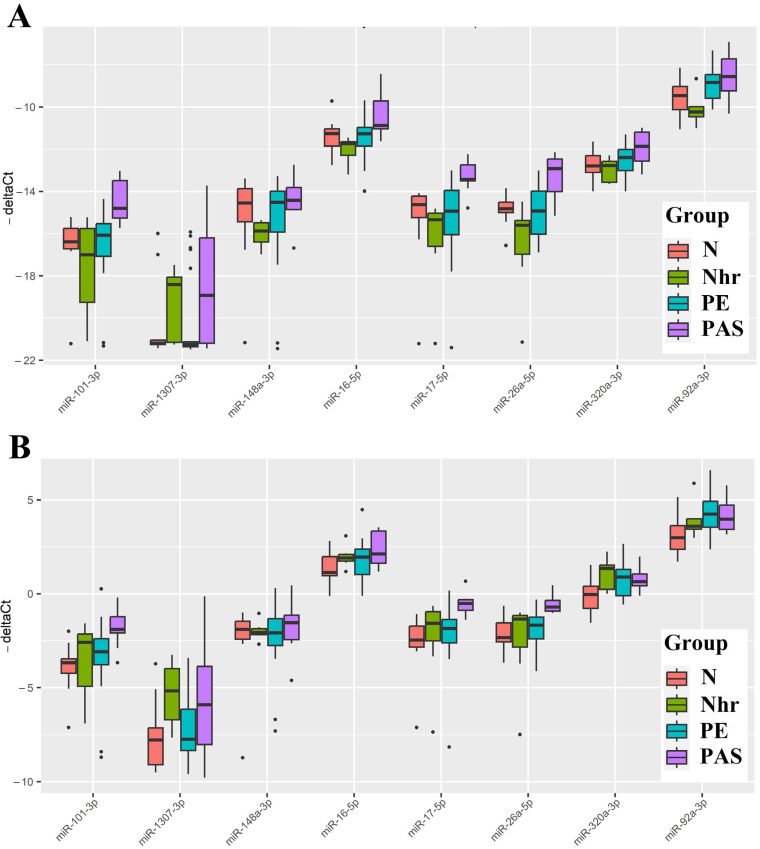
Box plot of miRNA content in native blood serum of women at 11–14 weeks of pregnancy in groups “N,” “Nhr,” “PE,” and “PAS.” “−ΔCt” Values were calculated using exogenous RNA UniSp6 (**A**). “−ΔCt” values were calculated relative to the content of endogenous miRNA hsa-let-7a-5p (**B**). The position of the median inside the box in the form of a horizontal line and outliers in the form of points are indicated.

From Figure 6 and Table 5, it can be inferred that the PAS group statistically significantly differed from the N group only in the elevated levels of hsa-miR-320a, hsa-miR-92a-3p, and hsa-miR-1307-3p among all the analyzed miRNAs. As miRNAs are secreted by cells not only as part of exosomes but also as part of lipoproteins [37], we analyzed the levels of hsa-miR-92a-3p, hsa-miR-320a, hsa-miR-101-3p, hsa-miR-26a-5p, hsa-miR-148a-3p, hsa-miR-1307-3p, hsa-miR-16-5p, and hsa-miR-17-5p in the native blood serum sample of the third patient cohort (Table 4). Two types of normalization were used to calculate the “−ΔCt” values: normalization to the exogenous RNA UniSp6 (Figure 7A, Table 6) and endogenous hsa-let-7a-5p (Figure 7B, Table 6).

In comparison with the results of quantitative assessment of miRNA in the exosomal fraction of blood serum from women at 11–14 weeks of pregnancy, we observed a statistically significant increase in the levels of hsa-miR-101-3p, hsa-miR-26a-5p, hsa-miR-16-5p, and hsa-miR-17-5p in the native blood serum of women in the PAS group relative to the N group (Table 6). We conclude that such increase in the levels of these miRNAs occurs in the non-exosomal fraction of blood serum. Notably, only hsa-miR-101-3p, hsa-miR-26a-5p, and hsa-miR-17-5p showed statistical significance in distinguishing the PAS group from all other groups (N, Nhr, PE) using two types of normalization: on UniSp6 or hsa-let-7a-5p. Hence, these miRNAs can be considered specific markers for PAS. The other three miRNAs—hsa-miR-92a-3p, hsa-miR-320a, and hsa-miR-16-5p—did not show statistically significant differences between the PAS group and the PE and/or Nhr groups (Table 6), indicating that they cannot be considered specific markers for PAS.

**Figure 8:**
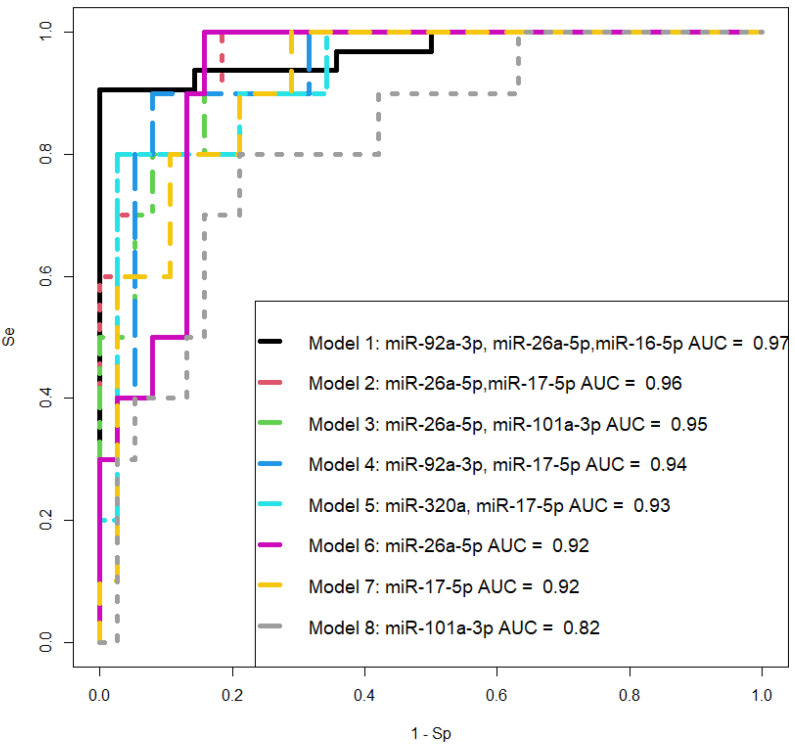
ROC curves of logistic regression models based on real-time PCR data comparing the PAS group and combined groups “N + Nhr + PE” for the content of miRNA in native blood serum of women at 11–14 weeks of pregnancy. Se—sensitivity, Sp—specificity.

The quantitative analysis of hsa-miR-92a-3p, hsa-miR-320a, hsa-miR-101-3p, hsa-miR-26a-5p, hsa-miR-148a-3p, hsa-miR-1307-3p, hsa-miR-16-5p, and hsa-miR-17-5p in the native blood serum of women at 11–14 weeks of pregnancy, using endogenous hsa-let-7a-5p as a normalizing RNA, was employed to develop logistic regression models for creating a non-invasive diagnostic system for PAS. In RStudio, optimal combinations of miRNAs associated with the presence of PAS in pregnant women were identified via stepwise inclusion and exclusion of each molecule in logistic regression models, where the dependent variable (response variable) was the presence or absence of PAS in pregnant women (0—combined groups N, Nhr, PE; 1—PAS group). The models presented in Figure 8 were selected, where all independent variables were statistically significant. The parameters of the models in Figure 8 are indicated in Table 7. Formulas 4 and 5, describing Models 2 and 3 in Figure 8, respectively, are provided below.

From Table 7, it can be inferred that logistic regression models 1, 2, and 3 have 100% sensitivity in detecting PAS in women during the first-trimester screening based on the quantitative analysis of the corresponding miRNAs in native blood serum. Since model 1 includes miR-92a-3p, the expression level of which was statistically significantly changed in both PE and PAS compared to normal pregnancy (Table 6), it cannot be considered as a specific marker for PAS, and therefore Model 1 was excluded from consideration.

(4) Formula for Model 2:

(4) $$\frac{1}{1+e^{-5.66-4.19x_{1}-2.92x_{2}}}$$

where *x*_1_—«−∆Ct» value for hsa-miR-26a-5p, *x*_2_—«−∆Ct» value for hsa-miR-17-5p;

(5) Formula for Model 3:

(5) $$\frac{1}{1+e^{-8.05-5.54x_{1}-1.99x_{2}}}$$

where *x*_1_—«−∆Ct» value for hsa-miR-26a-5p, *x*_2_—«−∆Ct» value for hsa-miR-101-3p.

Considering all 48 blood serum samples (Table 4), a reliable correlation of miRNA level with biochemical and instrumental analysis data at 11–14 GW were found using the non-parametric Spearman rank correlation method: hsa-miR-101-3p with NT (r = 0.34, *p* = 0.0188), hsa-miR-17-5p with UA(PI)MoM (r = −0.3, *p* = 0.038), hsa-miR-26a-5p with PLGF (r = 0.49, *p* = 0.025), hsa-miR-17-5p with PLGF (r = 0.36, *p* = 0.0327). At the same time, statistically significant correlations were found for UA(PI)MoM with PAPP-A (r = −0.33, *p* = 0.0242) and with PAPP-A(MoM) (r = −0.29, *p* = 0.043), for PLGF with PAPP-A (r = 0.37, *p* = 0.0264).

## 3. Discussion

In this study, the miRNA signature in placental tissues was analyzed both within the invasive region and outside this area, in the adjacent myometrial tissues, and in the blood plasma from women using deep sequencing. The obtained data were validated through quantitative real-time PCR to construct logistic regression models as a non-invasive diagnostic approach for differentiating various types of placenta accreta spectrum (PAS) in the third trimester of pregnancy. It was observed that the quantitative assessment of hsa-miR-320b, hsa-let-7d-5p, hsa-miR-629-5p, and hsa-miR-17-5p in the blood plasma of women allows for the statistically significant identification of PAS cases with high specificity (93–100%) and sensitivity (78–97%). These molecules were found to be differentially expressed in both placental and myometrial tissues of women with different types of PAS. The constructed logistic regression models can be considered as an additional diagnostic method alongside commonly used instrumental diagnostic approaches such as ultrasound (US) and magnetic resonance imaging (MRI). 

To formulate an individual management strategy for patients with PAS, involving the referral to a specialized hospital with a multidisciplinary team of surgeons, intensivists, neonatologists, and preparedness for blood transfusion in case of hemorrhage, it is imperative to diagnose this pregnancy complication in the first trimester. Due to the absence of precise biochemical and instrumental tests for detecting PAS during this gestational period, we conducted a retrospective study using deep sequencing and quantitative real-time PCR of cell-free miRNAs in the blood serum of women who underwent first-trimester screening and continued examination until the parturition with a clear diagnosis (physiological pregnancy or pre-eclampsia or PAS) at the Kulakov National Medical Research Center of Obstetrics, Gynecology, and Perinatology. The search for miRNA markers of placental invasion specifically in the exosomal fraction of blood serum was motivated by our previous findings [38] indicating a decrease in the concentration of identified miRNA markers for pre-eclampsia upon repeated cycles of freezing/thawing of the analyzed blood serum sample. The question of the miRNA stability in the body’s biological fluids has been thoroughly investigated by other researchers such as Coenen-Stass et al. [39]. Exosomes, being membrane-containing structures, protect the encapsulated miRNAs from degradation by extracellular RNases and facilitate the targeted delivery of miRNAs to specific cells and tissues [40]. We discovered that the most significant contributors to the differentiation of exosome fractions in the blood serum of women at 11–14 weeks of pregnancy with various types of PAS from combined groups of women with physiological pregnancy and pre-eclampsia, using principal component analysis, were miR-92a-3p, miR-320a, miR-101-3p, miR-26a-5p, miR-148a-3p, miR-1307-3p, miR-16-5p, and miR-17-5p based on deep-sequencing data. Among these, statistically significant differences between the PAS group and all other comparable groups were identified only for miR-320a, miR-92a-3p, and miR-1307-3p based on quantitative real-time PCR data.

However, the isolation of exosomes for identifying cases of placenta accreta spectrum (PAS) using marker mRNA is an additional time-consuming and costly method, which may not be feasible given the high patient volume during screening studies. Therefore, we decided to analyze exosome mRNA markers for PAS in the native blood serum of pregnant women at 11–14 weeks of gestation using quantitative real-time PCR. In comparison with the analysis of the exosome fraction of blood serum, we observed a significant increase in the levels of miR-101-3p, miR-26a-5p, miR-16-5p, and miR-17-5p, in addition to changes in miR-92a-3p and miR-320a, in the native blood serum of women from the PAS group compared to the group of women with physiological pregnancies. This increase in the levels of these mRNA markers occurs in the non-exosome fraction, possibly as part of very-low-density lipoproteins (VLDL), low-density lipoproteins (LDL), or high-density lipoproteins (HDL). For example, other researchers demonstrated the presence of some of the analyzed here miRNAs in blood lipoproteins, namely miR-16-5p in VLDL and HDL, miR-17-5p and miR-26a-5p in HDL [37], dependent on miRNA sequence motifs. Mechanistically, it was found that HDL-miRNAs have roles in metabolic homeostasis and angiogenesis, whereas targets for LDL-miRNAs were enriched in pathways related to inflammation, immune system function, and different cardiomyopathies.

When calculating “−ΔCt” values for each analyzed miRNA, any of the RNA species was used as a reference: either UniSp6, introduced into the sample during the reverse transcription stage according to the Qiagen’s recommendation, or the endogenous let-7a-5p, exhibiting a stable high expression level in the exosomal fraction of blood serum and native blood serum of women at 11–14 weeks of pregnancy according to our deep-sequencing data. The use of UniSp6 allows for accounting for variations in reverse transcription and PCR efficiency but does not account for possible mRNA degradation due to extracellular RNases during repeated sample freezing/thawing cycles, unlike the use of endogenous let-7a-5p. The presence of let-7a-5p in exosomes, as identified in the current study, and in VLDL, as reported by Guido Rossi-Herring [37], when used as an endogenous reference RNA, would consider detrimental processes affecting the concentration of analyzed miRNA in exosomes and the non-exosomal fraction of blood serum ex vivo.

It is important to note that miR-101-3p, miR-26a-5p, and miR-17-5p were found to be the most specific markers for PAS, as statistically significant differences in their expression levels were identified in the PAS group compared to all other comparison groups (physiological pregnancy, high risk of developing pre-eclampsia according to Astraia during the first pregnancy screening without clinical manifestations of pre-eclampsia after 20 weeks of pregnancy, development of early and late pre-eclampsia) using two types of normalization: on UniSp6 or let-7a-5p. Meanwhile, other miRNA associated with PAS (miR-92a-3p, miR-320a, miR-16-5p) did not significantly differentiate the PAS group from the pre-eclampsia and/or high-risk group for developing pre-eclampsia without clinical manifestations after 20 weeks of pregnancy. Therefore, the latter cannot be considered specific markers for PAS.

The potential role of miR-92a-3p, miR-320a, and miR-16-5p in the pathogenesis of pre-eclampsia (PE) has been studied in several works, revealing that (i) intravascular inflammation occurs in PE as a sequence of Th1 polarization [41,42] through the targeting of GATA3 by upregulated miR-92a-3p, contained in vesicles of activated NK cells [43]; (ii) miR-320a overexpression observed in PE inhibits trophoblast cell invasion and causes anomalous placentation by targeting estrogen-related receptor-gamma [44], IL-4 [45], and IGF-1R [46]; (iii) in a PE rat model, the upregulation of miR-16-5p directly downregulates IGF-2 and provides inhibition of trophoblast cell viability and migration [47]. Thus, the involvement of the same miRNA molecules in different diseases necessitates the search for a unique combination of marker miRNAs that can differentiate one pregnancy complication from another. Therefore, when selecting logistic regression models for diagnosing PAS at 11–14 weeks of gestation, we relied on the following criteria: the combination of molecules should include any or all the miRNAs miR-101-3p, miR-26a-5p, and miR-17-5p; all model parameters must be significant, and the model should have high specificity and sensitivity. These criteria are met by two models we developed: the combination of miR-26a-5p and miR-17-5p, and the combination of miR-26a-5p and miR-101-3p, both of which exhibit 100% sensitivity in detecting PAS in women during the first pregnancy screening through their quantitative analysis in native blood serum using real-time PCR.

It is important to note that the circulating miRNA markers for PAS identified in this study during the first trimester of pregnancy are differentially expressed in the placenta within the area of invasion and in the adjacent myometrium, specifically in the case of invasive forms (increta and percreta) relative to the adherent form (creta) in the third trimester of pregnancy at the time of delivery, as per deep-sequencing data. Comparing areas of the placenta outside the invasive region led us to hypothesize that for placental invasion into the myometrium, molecular and biological changes in the myometrial tissue are necessary. This assumption is based on the observation that the intersection of differentially expressed miRNA lists in areas of the placenta outside the invasion site, in the case of increta and percreta relative to creta, is found for 70–80% of all miRNAs. And more pronounced quantitative and qualitative changes in the miRNA signature are observed in the area of trophoblast invasion in both placental tissue and adjacent myometrium from women with placenta percreta. In other words, for the invasive type of PAS, there are fundamental changes in the miRNA signature in the placenta, similar between increta and percreta but distinct from PAS adherent form (creta); the invasion of such altered placenta into the myometrial tissue occurs only if there are changes in the myometrium itself, most pronounced in the case of percreta. These findings are consistent with those of other researchers who adhere to the concept of a primary deciduomyometrium defect in PAS that impacts the formation of the migratory and invasive phenotype of interstitial and endovascular extravillous trophoblast cells [13,14].

When analyzing experimentally validated target genes of identified here miRNAs differentially expressed in the placenta and myometrium within the PAS site, signaling pathways involving growth factors, glypicans, cell adhesion proteins, integrins, interleukins, and chemokines were identified. These pathways are responsible for processes such as cell adhesion, proliferation, migration, angiogenesis, inflammation, and apoptosis. These data align with discussions in published articles on pathways that stimulate trophoblast invasion [12,48].

A distinctive feature of invasive PAS, as observed through instrumental research methods and macroscopic examination of the uterine surface, is uteroplacental vascular changes in the accreta area resulting from both neovascularization and/or increased infiltration of deep uterine vessels (radial and even the arcuate arteries) by extravillous trophoblasts (EVT) [1]. The role in angiogenesis of the identified here miRNAs as the markers of PAS in the first trimester of pregnancy has been demonstrated by numerous scientific teams. Hypoxia-responsive hsa-miR-101-3p, known as angiomiR, regulate angiogenesis by targeting cullin 3 thereby promoting Nrf2 nuclear accumulation and causing heme oxygenase-1 induction, VEGF expression, and nitric oxide production [49], or by targeting c-Met [50]—a receptor for hepatocyte growth factor (HGF), which is one of the key molecules that stimulate endothelial cells to proliferate and migrate via the upregulation of VEGF and its receptor KDR [51] as well as metalloproteinases [52] to degrade extracellular matrix for vascular growth. In addition, the inverse correlation between the expression of the miR-320a and the HGF gene was found [50]. Different studies demonstrated the suppressor function of miR-320a in cell invasion and angiogenesis in ovary cancer [53], hepatocellular carcinoma [54,55], and endometrial cancer [56]. The anti-inflammatory effect and promotion of the angiogenesis in the skeletal muscle injury model were found for miR-320a and miR-26a via reduction of the protein expression of their target genes—PTEN and TLR3, respectively [57]. Enriched by miR-17-5p exosomes from endothelial progenitor cell decrease cell apoptosis, increase microvessel density and capillary angiogenesis as well as promote muscle structural integrity in a diabetic hind-limb ischemia mode through increasing the levels of PI3K and phosphorylated Akt [58]. The participation of miR-92a-3p in exosome-mediated angiogenesis was found in retinoblastoma by targeting transcription factor KLF2 [59] which is able to modulate tumor proliferation and metastasis [60]. The significant correlations of hsa-miR-17-5p expression level with uterine artery pulsatility index, and hsa-miR-26a-5p and hsa-miR-17-5p with PLGF revealed in the present study prove the important role of these PAS miRNA markers in angiogenesis.

## 4. Materials and Methods

### 4.1. Patients

All patients (Table 1, Table 2 and Table 4) enrolled to investigation were admitted to the National Medical Research Center for Obstetrics, Gynecology, and Perinatology, named after the Academician V.I. Kulakov of Ministry of Healthcare of the Russian Federation for management of pregnancy and delivery, and signed informed consent to participate in the study; the study was approved by the Ethics Committee of the Center. Clinical and biochemical blood tests, ultrasound examination of the pelvic and fetal organs, fetoplacental blood flow dopplerometry, cardiotocography, blood pressure measurement, the determination of protein levels in urine, and concentrations of PLGF, sFlt-1, PAPP-A, and β-HCG in blood serum using diagnostic test systems were carried out for each patient. The criteria for non-inclusion in the study were the onset of pregnancy via assisted reproductive technologies, multiple pregnancy, and fetal aneuploidy.

### 4.2. RNA Isolation from Blood Plasma or Serum

A total of 200 µL of blood plasma or serum, purified from cells and cell debris via stepwise centrifugations at 300× *g* for 20 min and at 16,000× *g* for 10 min, were used for RNA isolation using an miRNeasy Serum/Plasma kit (Qiagen, Hilden, Germany) according to the manufacturer’s protocol.

### 4.3. RNA Isolation from Placenta or Myometrium Tissue

Placenta or myometrium tissue samples were taken for research no later than 10 min after delivery as shown in Figure 1A, and immediately frozen in liquid nitrogen for subsequent storage at −80 °C. Total RNA was extracted from 20 to 40 mg of tissue using the miRNeasy Micro Kit (Qiagen, Hilden, Germany) followed by the RNeasy MinElute Cleanup Kit (Qiagen, Hilden, Germany) according to the manufacturer’s protocol. The RNA concentration was measured using a Qubit fluorimeter 3.0 (Life Technologies, Petaling Jaya, Malaysia). The sample quality of the total RNA was examined using the Agilent Bioanalyzer 2100 (Agilent, Waldbronn, Germany) and the RNA 6000 Nano Kit (Agilent Technologies, Santa Clara, CA, USA). Total RNA samples with a 28S/18S ribosomal RNA ratio equal to 1.5–1.8 were used for further studies.

### 4.4. RNA Isolation from Blood Serum Exosomes

A total of 200 µL of blood serum, purified from cells and cell debris via stepwise centrifugations at 300× *g* for 20 min and at 16,000× *g* for 10 min, were used for RNA isolation using an exoRNeasy Midi Kit (Qiagen, Hilden, Germany) according to the manufacturer’s protocol.

### 4.5. miRNA Deep Sequencing

cDNA libraries were synthesized using 6 µL of total RNA column eluate (miRNeasy Serum/Plasma Kit) extracted from 200 µL of blood plasma, 6 µL of total RNA column eluate (exoRNeasy Midi Kit) extracted from 200 µL of blood serum, and 500 ng of total RNA from placenta or myometrium tissue, using the NEBNext^®^ Multiplex Small RNA Library Prep Set for Illumina^®^ (Set11 and Set2, New England Biolab^®^, Frankfurt am Main, Germany, cat. nos. E7300S and E7580S), amplified for 19 cycles in case of blood plasma samples, 21 PCR cycles in case of blood serum exosomes, and 14 PCR cycles for placenta or myometrium samples, purified by QIAQuick PCR Purification Kit (Qiagen, Hilden, Germany), and 6% polyacrylamide gel electrophoresis for isolation of 136–150 bp bands corresponding to the adapter-ligated miRNAs, and sequenced using the NextSeq 500 platform (Illumina, San Diego, CA, USA, cat. no. SY-415-1001). The adapters were removed using Cutadapt. All trimmed reads shorter than 16 bp and longer than 55 bp were filtered, and only reads with a mean quality higher than 15 were retained. The remaining reads were mapped to the GRCh38.p15 human genome and miRBase v21 using the bowtie aligner [61]. Aligned reads were counted using the featureCount tool from the Subread package [62] and the fracOverlap 0.9 option; thus, the whole read was forced to have a 90% intersection with sncRNA features. Differential expression analysis of the sncRNA count data was performed using the DESeq2 package [63].

### 4.6. Reverse Transcription and Quantitative Real-Time PCR

Two microliters of total RNA column eluate (miRNeasy Serum/Plasma Kit, Qiagen, Hilden, Germany) or total RNA column eluate (exoRNeasy Midi Kit) was converted into cDNA in accordance with the miRCURY LNA RT Kit (Qiagen, Hilden, Germany) protocol; then, the sample volume was diluted 1:60, and cDNA was amplified in accordance with the miRCURY LNA miRNA SYBR Green PCR (Qiagen, Hilden, Germany) protocol using the miRCURY LNA miRNA PCR Assay (cat. no. 339306) in a StepOnePlus^TM^ thermocycler (Applied Biosystems, Waltham, MA, USA). The relative expression of miRNA in the sample was determined by the ∆Ct method, using UniSp6 or hsa-let-7a-5p as the reference RNA.

### 4.7. Statistical Analysis of the Obtained Data

For statistical processing, scripts written in R language [62] and RStudio [64] were used. The correspondence of the analyzed parameters to the normal distribution law was assessed via the Shapiro–Wilk test. When the distribution of data was different from normal, the Mann–Whitney test for paired comparison was used. Since both quantitative and qualitative characteristics were analyzed, a rank-order correlation analysis was performed using Spearman’s non-parametric correlation test. The 95% confidence interval for the correlation coefficient was determined using Fisher transformation. The value of the threshold significance level (*p*) was taken as being equal to 0.05.

## 5. Conclusions

Two logistic regression models have been developed for diagnosing PAS in women at 11–14 weeks of pregnancy by quantifying cell-free miRNAs hsa-miR-101-3p, hsa-miR-26a-5p, and hsa-miR-17-5p circulating in the peripheral blood, which are crucial for improving maternal and perinatal outcomes. These models demonstrated 100% sensitivity in detecting PAS during the first pregnancy screening. Hsa-miR-101-3p, hsa-miR-26a-5p, and hsa-miR-17-5p were identified as specific markers for PAS but not for other pregnancy complications such as early- and late-onset pre-eclampsia, and significantly correlated with nuchal translucency thickness (in the case of hsa-miR-101-3p), uterine artery pulsatility index (in the case of hsa-miR-17-5p), and PLGF (in the case of hsa-miR-26a-5p and hsa-miR-17-5p). A limitation of the developed diagnostic method is the small number of training samples due to the lack of a complete examination of the majority of pregnant women within one clinical center, namely the National Medical Research Center for Obstetrics, Gynecology, and Perinatology of Ministry of Healthcare of the Russian Federation, and the admission of pregnant women with PAS to this center only for delivery without the ability to analyze biological material (peripheral blood serum) donated at another clinical center during the first-trimester screening and vice versa. In connection with the emerging new screening method in the context of this study, pregnant women with an identified high risk of PAS by this method in the National Medical Research Center for Obstetrics, Gynecology, and Perinatology of Ministry of Healthcare of the Russian Federation will be offered to undergo further management of pregnancy and delivery in the same Center, which will expand the training set followed by validation of method efficiency on an independent test set with the integration of clinical and biochemical PAS markers to improve the accuracy of timely PAS diagnosis. 

## Figures and Tables

**Table 1:** Clinical Characteristics of the First Cohort of Patients.

No	Age	Delivery Time (Weeks)	PAS	Blood Plasma Sample *	Placenta Sample with Pathologic Trophoblast Invasion *	Placenta Sample with Normal Trophoblast Invasion *	Myometrium Sample Adjacent to Placenta with Pathologic Trophoblast Invasion *
1	33	34.4	Absent	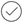			
2	29	36.5	Absent	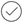			
3	29	38.3	Absent	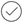			
4	37	38.2	Absent	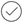			
5	30	39	Absent	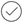			
6	30	34.1	Creta	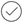			
7	42	35.4	Creta	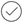	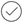		
8	35	36.4	Creta	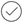	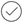		
9	35	36	Creta	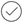	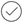		
10	38	36	Creta	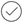	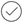		
11	40	34.2	Creta	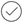	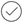	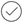	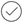
12	42	35.2	Creta	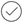	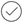	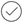	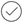
13	28	35	Creta	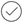	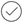	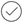	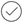
14	39	37	Increta	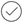	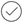		
15	34	35.3	Increta	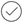	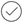		
16	34	34.6	Increta	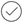	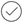	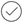	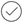
17	33	35	Increta	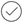	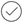	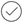	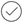
18	35	34.2	Increta	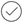	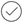	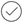	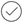
19	32	36.1	Increta	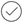	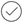	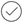	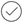
20	37	35.2	Percreta	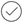			
21	37	36	Percreta	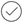	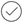		
22	37	33	Percreta	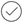	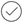	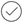	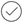
23	32	35.1	Percreta	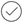	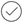	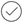	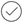
24	37	35.4	Percreta	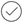	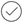	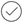	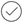

* 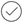 Checkmark indicates the analysis of miRNA levels in the sample using deep sequencing.

**Table 2:** Clinical Characteristics of the Second Cohort of Patients.

Group Name	Group Size	Scar on the Uterus	Placenta Previa	Delivery Time (Weeks) *
Norma (pregnancy without complications)	14	yes	no	37.5 (34.3; 39.2)
Creta	9	yes	yes	36.4 (35; 37.4)
Increta	16	yes	yes	34.6 (33.1; 36.3)
Percreta	7	yes	yes	33 (32; 33)

* Data are presented as the median (Me) and quartiles Q1 and Q3 in the format: Me (Q1; Q3).

**Table 3:** Parameters of the logistic regression models in Figure 4.

Model		Estimate (95% CI)	Wald	*p*-Value	OR (95% CI)	Se	Sp
**1**	**(Intercept)**	12.4342 (5.8431; 25.1735)	2.6862	0.0072	251,251.6477 (344.8485; 85,646,274,941.2615)	0.97	0.93
	**miR-320b**	−2.6129 (−5.2514; −0.9477)	−2.504	0.0123	0.0733 (0.0052; 0.3876)		
	**miR-92a-3p**	−2.1527 (−4.8586; −0.5747)	−2.0715	0.0383	0.1162 (0.0078; 0.5629)		
**2**	**(Intercept)**	9.7122 (4.807; 18.8667)	2.8577	0.0043	16,518.5771 (122.3664; 156,204,305.6105)	0.78	1
	**miR-320b**	−4.0078 (−7.9435; −1.83)	−2.6992	0.007	0.0182 (0.0004; 0.1604)		
	**let-7d-5p**	−0.6938 (−1.4087; −0.1879)	−2.3393	0.0193	0.4996 (0.2445; 0.8287)		
**3**	**(Intercept)**	11.9713 (5.7515; 21.3935)	3.0688	0.0021	158,149.4506 (314.6611; 1,954,609,961.5401)	0.88	0.93
	**miR-17-5p**	−0.7119 (−1.3684; −0.2466)	−2.5573	0.0105	0.4907 (0.2545; 0.7815)		
	**miR-25-3p**	−0.5773 (−1.0826; −0.2314)	−2.7482	0.006	0.5614 (0.3387; 0.7934)		
**4**	**(Intercept)**	5.4891 (2.9334; 9.5854)	3.3713	<0.001	242.042 (18.7919; 14,550.8746)	0.88	0.86
	**miR-320b**	−2.8885 (−5.1368; −1.4507)	−3.1781	0.0015	0.0557 (0.0059; 0.2344)		
**5**	**(Intercept)**	9.5615 (4.6005; 16.6831)	3.1352	0.0017	14,206.4468 (99.5329; 17,593,816.9241)	0.78	1
	**miR-629-5p**	−0.4622 (−0.8576; −0.1463)	−2.61	0.0091	0.6299 (0.4242; 0.8639)		
	**miR-17-5p**	−0.5663 (−1.1491; −0.1557)	−2.2973	0.0216	0.5676 (0.3169; 0.8558)		
**6**	**(Intercept)**	8.6533 (4.3535; 15.0105)	3.2633	0.0011	5,729.094 (77.7512; 3,303,537.1587)	0.91	0.86
	**miR-92a-3p**	−2.2867 (−3.9974; −1.1017)	−3.1624	0.0016	0.1016 (0.0184; 0.3323)		
**7**	**(Intercept)**	5.891 (2.3393; 11.7884)	2.5906	0.0096	361.7674 (10.3736; 131,711.3987)	0.94	0.71
	**miR-17-5p**	−0.5333 (−1.0924; −0.1761)	−2.4108	0.0159	0.5866 (0.3354; 0.8385)		

**Table 4:** Clinical characteristics of the third cohort of patients.

Sample ID	Diagnose at Delivery, Group Name	Biochemical Data						Ultrasound Data			
		1st Trimester Screening, Peripheral Blood Sampling, GW	PAPP-A (0.7–6.0 IU/L)	PAPP-A (0.5–2.0 MoM)	b-hCG (50.0–55.0 IU/mL)	b-hCG (0.5–2.0 MoM)	PLGF (21.9–71.2 пг/мл)	Crown Rump Length, CRL (43.0–84.0 mm)	Nuchal Translucency Thickness, NT (1.6–1.7 mm)	Uterine Artery Pulsatility Index, UA (PI), MoM	Uterine Artery Pulsatility Index, UA (Pi), 0.9–2.6 (5th and 95th Percentiles)
1	late-onset pre-eclampsia, PE	12.1	1.72	0.65	51.1	0.94	23.8	57.4	1.3	0.75	1.27
6		12.2	1	0.5	53.6	1.64	15.8	58	1.7	0.94	1.6
7		12.1	4.3	1.7	27.9	0.57	15.79	60.5	1.9	1.21	2.06
11		12.5	1.92	0.64	40.5	0.81	20.9	63.2	1.91	1.4	2.35
12		12	4.27	1.21	28.1	0.46	8.6	58.7	1.5	0.73	1.28
14		12.5	3.13	0.65	27.8	0.45	24.01	63.3	1.4	1.33	2.32
16		12.5	2.96	1.03	35.6	0.79	15.6	64	2	0.4	0.65
17		12.1	0.61	0.4	28.3	0.76	15.8	58.6	1.5	1.45	2.28
18		11.6	1.63	0.55	42.9	0.69	9.34	55.1	1.5	1.04	1.86
19		12.4	5.01	2.65	28.1	0.51	26.5	57.6	1.3	0.81	1.41
21	early-onset pre-eclampsia, PE	12.1	1.86	0.9	54	1.09		57	1.6	2.07	3.5
22		12.1	0.8	0.46	36	0.6		50.6	1.76	1.18	2.09
23		12.2	3.36	1.17	45.9	0.92	11.7	60.2	2.9	1.22	2.04
24		11.2	1.96	1.04	94.3	1.66		50	1.07	1.2	2.12
25		11.6	6.2	2.96	43.8	0.76	10	56	1.45	1.03	1.83
26		12.1	2.06	0.79	48.8	1.05	9.2	60.3	1.7	1.56	2.6
27		12.3	0.86	0.45	15.6	0.36		59	1.9	1.2	2
28		11.6	2.47	2.27	34.3	0.98	12.84	57	1.3	1.01	1.59
29		12.4	2.9	1	56.8	1.1	18	62	1.3	0.79	1.31
30		12.4	1.71	0.64	31.3	0.7	6.3	62	1.5	0.98	1.68
31		12	2.09	0.83	16.9	0.33	16	57.7	1.7	0.33	1.686
33	physiological pregnancy, N	12.5	2.28	0.86	89.7	2.17	19.7	63.9	2.2	0.7	1.12
34		12.1	2	0.71	89.8	2.25	16.39	66	1.6	1.09	1.7
35		13.4	2.57	0.83	62.2	1.81	32.7	74.7	1.8	1.19	1.77
39		12	3.02	1.52	66	1.32	16.11	54	1.1	1.028	1.75
41		13	3.1	0.88	61	1.26	26.68	67.5	1.6	0.28	0.44
43		12.2	1.62	0.52	86.7	1.77	14.7	58	1.2	1.14	1.95
44		13	3.42	1.3	56.6	1.44	15.97	66	1.21	1.02	1.6
46		12	3.011	1.23	64.2	1.15	15.49	54.5	1.2	1.002	1.77
47		12.6	2.9	0.61	52.3	1.06	32.52	65	1.17	1.3	2.15
49		12	6.9	3.15	58.8	1.105	26.2	55	1.3	0.88	1.49
51	high risk of pre-eclampsia according to Astraia but no pre-eclampsia at delivery, Nhr	11.2	1.05	0.41	25.5	0.36		50	1	1.33	2.48
52		13	3.38	0.68	39.8	0.74		68	2	1.15	1.92
53		11.6	1.55	1.32	23.7	0.717	12.7	59.7	1.9	1.4	2.17
54		12.1	2.1	1.07	89.8	2.5		56	1.1	1.02	1.68
55		12.2	2.25	0.97	23.2	0.48	19.99	57.6	1.6	1.19	2.02
56		11.6	2.27	1.23	114.7	2.3		53	1.29	1.05	1.8
58		12.1	4.18	1.8	51.7	1.02		57	1.9	0.81	1.36
110	Placenta percreta, PAS	12.2	13.7	4.6	101.21	2.047	37.7	61.9	1.7	1.069	1.75
111	Placenta increta, PAS	12.5	5.117	1.983	47	0.872	25.6	61	1.3	0.49	0.855
112	Placenta creta, PAS	12	4.46	2.145	38.14	0.69	22.8	53	1.2	0.981	1.715
113	Placenta increta, PAS	12.3	1.891	0.995	35	0.86		59.6	1.8	0.845	1.365
114	Placenta percreta, PAS	12.6	3.257	1.043	65.17	1.428	23.3	65	1.9	0.552	0.89
115	Placenta percreta, PAS	13.1	2.5	1.151	28.06	0.9	56.2	70	2	0.999	1.475
116	Placenta increta, PAS	12.0	5.84	2.021	223.6	3.97		57.9	1.2	1.164	1.995
117	Placenta increta, PAS	13.0	8.6	2.8	53	1.309	34.8	68	1.5	0.485	0.755
118	Placenta increta, PAS	12.1	3.838	1.562	45.84	0.865		56.8	1.6	1.312	2.21
119	Placenta percreta, PAS	12.4	2.252	0.989	86.85	2.06		61.7	1.9	0.753	1.22

**Table 5:** Comparison of groups “N” and “PAS” by the miRNA “−ΔCt” value plotted as a box diagram in Figure 6.

miRNA	Group	Me	Q1	Q3	*p*-Value
miR-101-3p	N	−2.62	−2.89	−2.31	0.25
	PAS	−2.69	−3.13	−2.52	
miR-16-5p	N	0.96	0.75	1.08	0.09
	PAS	0.67	0.26	0.86	
miR-17-5p	N	−0.53	−0.82	−0.44	0.08
	PAS	−0.89	−0.93	−0.82	
miR-26a-5p	N	0.43	0.26	0.64	0.53
	PAS	0.37	0.15	0.67	
miR-320a	N	−0.54	−0.85	−0.28	0.006
	PAS	−0.13	−0.18	0.43	
miR-92a-3p	N	0.58	0.45	0.95	<0.001
	PAS	2.05	1.68	2.28	
miR-1307-3p	N	−5.85	−6.41	−5.15	0.04
	PAS	−4.84	−5.14	−4.72	
miR-148a-3p	N	−3.26	−4.69	−2.85	0.44
	PAS	−3.71	−3.99	−3.52	

**Table 6:** Pairwise comparison of third patient cohort groups listed in Table 4 by miRNA “−ΔCt” value, presented as a box diagram in Figure 7.

**Normalization to UniSp6**								
**miRNA**	**Group**	**Me**	**Q1**	**Q3**	***p*-Value**			
					**N**	**Nhr**	**PE**	**PAS**
**miR-101-3p**	**N**	−16.39	−16.72	−15.75	-	0.53	0.54	<0.001
	**Nhr**	−17	−19.25	−15.75	0.53	-	0.43	<0.001
	**PE**	−16.08	−17.07	−15.53	0.54	0.43	-	<0.001
	**PAS**	−14.81	−15.26	−13.48	<0.001	<0.001	<0.001	-
**miR-16-5p**	**N**	−11.26	−11.85	−11.04	-	0.06	0.98	0.02
	**Nhr**	−11.74	−12.29	−11.64	0.06	-	0.08	<0.001
	**PE**	−11.27	−11.85	−10.96	0.98	0.08	-	0.01
	**PAS**	−10.87	−11.03	−9.71	0.02	<0.001	0.01	-
**miR-17-5p**	**N**	−14.63	−15.24	−14.23	-	0.07	0.85	<0.001
	**Nhr**	−15.35	−16.6	−15.03	0.07	-	0.17	<0.001
	**PE**	−14.93	−16.05	−13.95	0.85	0.17	-	<0.001
	**PAS**	−13.44	−13.5	−12.75	<0.001	<0.001	<0.001	-
**miR-26a-5p**	**N**	−14.82	−15	−14.51	-	0.02	0.88	0.002
	**Nhr**	−15.6	−16.98	−15.38	0.02	-	0.08	<0.001
	**PE**	−14.93	−16.02	−13.98	0.88	0.08	-	<0.001
	**PAS**	−12.92	−14.01	−12.46	0.002	<0.001	<0.001	-
**miR-320a-3p**	**N**	−12.79	−13.11	−12.31	-	0.88	0.39	0.02
	**Nhr**	−12.78	−13.57	−12.58	0.88	-	0.09	0.01
	**PE**	−12.39	−13.01	−12.01	0.39	0.09	-	0.07
	**PAS**	−11.86	−12.56	−11.2	0.02	0.01	0.07	-
**miR-92a-3p**	**N**	−9.46	−10.13	−9.03	-	0.13	0.12	0.05
	**Nhr**	−10.23	−10.46	−9.98	0.13	-	0.001	0.009
	**PE**	−8.84	−9.59	−8.46	0.12	0.001	-	0.24
	**PAS**	−8.56	−9.23	−7.72	0.05	0.009	0.24	-
**miR-1307-3p**	**N**	−21.19	−21.25	−21.05	-	0.31	0.34	0.31
	**Nhr**	−18.41	−21.15	−18.06	0.31	-	0.14	0.60
	**PE**	−21.24	−21.35	−21.13	0.34	0.14	-	0.09
	**PAS**	−18.92	−21.2	−16.2	0.31	0.60	0.09	-
**miR-148a-3p**	**N**	−14.55	−15.44	−13.87	-	0.07	0.63	0.73
	**Nhr**	−15.88	−16.4	−15.48	0.07	-	0.10	0.003
	**PE**	−14.52	−15.93	−13.99	0.63	0.10	-	0.30
	**PAS**	−14.42	−14.86	−13.81	0.73	0.003	0.30	-
**Normalization to Hsa-Let-7a-5p**								
**miRNA**	**Group**	**Me**	**Q1**	**Q3**	***p*-Value**			
					**N**	**Nhr**	**PE**	**PAS**
**miR-101-3p**	**N**	−3.68	−4.24	−3.46	-	0.36	0.12	<0.001
	**Nhr**	−2.59	−4.93	−2.14	0.36	-	0.95	0.05
	**PE**	−3.09	−3.78	−2.39	0.12	0.95	-	0.01
	**PAS**	−1.9	−2.09	−1.22	<0.001	0.05	0.01	-
**miR-16-5p**	**N**	1.13	0.96	1.98	-	0.16	0.28	0.02
	**Nhr**	1.91	1.74	2.09	0.16	-	0.83	0.41
	**PE**	1.96	1.03	2.38	0.28	0.83	-	0.17
	**PAS**	2.12	1.62	3.34	0.02	0.41	0.17	-
**miR-17-5p**	**N**	−2.46	−2.84	−1.72	-	0.41	0.21	<0.001
	**Nhr**	−1.58	−2.51	−0.95	0.41	-	0.53	0.009
	**PE**	−1.85	−2.62	−1.37	0.21	0.53	-	<0.001
	**PAS**	−0.51	−0.89	−0.31	<0.001	0.009	<0.001	-
**miR-26a-5p**	**N**	−2.33	−2.56	−1.55	-	0.73	0.23	<0.001
	**Nhr**	−1.35	−2.83	−1.15	0.73	-	0.91	<0.001
	**PE**	−1.67	−2.4	−1.24	0.23	0.91	-	<0.001
	**PAS**	−0.71	−0.92	−0.35	<0.001	<0.001	<0.001	-
**miR-320a-3p**	**N**	−0.04	−0.78	0.4	-	0.05	0.11	0.05
	**Nhr**	1.35	0.24	1.52	0.05	-	0.29	0.60
	**PE**	0.9	−0.1	1.3	0.11	0.29	-	0.88
	**PAS**	0.65	0.43	1.05	0.05	0.60	0.88	-
**miR-92a-3p**	**N**	2.99	2.37	3.64	-	0.10	0.03	0.03
	**Nhr**	3.6	3.44	3.99	0.10	-	0.46	0.53
	**PE**	4.25	3.55	4.93	0.03	0.46	-	1
	**PAS**	3.97	3.43	4.72	0.03	0.53	1	-
**miR-1307-3p**	**N**	−7.78	−9.1	−7.14	-	0.04	0.41	0.19
	**Nhr**	−5.17	−6.71	−3.98	0.04	-	0.03	0.60
	**PE**	−7.74	−8.34	−6.15	0.41	0.03	-	0.30
	**PAS**	−5.91	−8.02	−3.87	0.19	0.60	0.30	-
**miR-148a-3p**	**N**	−1.9	−2.42	−1.47	-	0.96	0.95	0.48
	**Nhr**	−2.07	−2.17	−1.86	0.96	-	1	0.36
	**PE**	−2.08	−2.76	−1.33	0.95	1	-	0.41
	**PAS**	−1.54	−2.44	−1.14	0.48	0.36	0.41	-

**Table 7:** Parameters of logistic regression models in Figure 8.

Models	Estimate (95% CI)	Wald	*p*-Value	OR(95% CI)	Se	Sp
(Intercept)	12.264 (3.532; 27.813)	2.149	0.032	211,919.348 (34.208; 1.2 × 10^12^)	**Model 1**	
miR-92a-3p	−4.955 (−10.911; −1.566)	−2.246	0.025	0.007 (0.00001; 0.209)	1	0.84
miR-26a-5p	5.093 (2.068; 10.91)	2.355	0.019	162.828 (7.905; 54,728.702)		
miR-16-5p	5.584 (1.896; 12.356)	2.267	0.023	266.031 (6.658; 232,425.187)		
(Intercept)	5.661 (1.863; 12.559)	2.157	0.031	287.563 (6.445; 284,717.908)	**Model 2**	
miR-26a-5p	4.189 (1.259; 9.276)	2.157	0.031	65.939 (3.523; 10,674.696)	1	0.82
miR-17-5p	2.921 (0.646; 6.887)	1.916	0.055	18.564 (1.908; 979.604)		
(Intercept)	8.049 (2.747; 17.86)	2.193	0.028	3129.605 (15.589; 5.7 × 10^7^)	**Model 3**	
miR-26a-5p	5.538 (2.059; 12.068)	2.274	0.023	254.057 (7.835; 174,165.021)	1	0.84
miR-101-3p	1.989 (0.502; 4.585)	2.012	0.044	7.308 (1.652; 98.023)		
(Intercept)	9.303 (2.248; 19.251)	2.21	0.027	10,967.88 (9.473; 2.3 × 10^8^)	**Model 4**	
miR-92a-3p	−1.451 (−3.193; −0.173)	−1.941	0.052	0.234 (0.041; 0.841)	0.9	0.9211
miR-17-5p	4.333 (1.956; 8.034)	2.872	0.004	76.151 (7.071; 3084.714)		
(Intercept)	4.152 (1.217; 8.272)	2.386	0.017	63.567 (3.378; 3911.935)	**Model 5**	
miR-320a-3p	−1.477 (−3.168; −0.148)	−1.973	0.048	0.228 (0.042; 0.862)	0.8	0.9737
miR-17-5p	4.108 (1.878; 7.607)	2.899	0.004	60.854 (6.539; 2013.226)		
(Intercept)	2.256 (0.321; 4.915)	1.974	0.048	9.544 (1.378; 136.287)	**Model 6**	
miR-26a-5p	3.389 (1.483; 6.373)	2.773	0.006	29.638 (4.405; 585.998)	1	0.8421
(Intercept)	1.628 (−0.009; 3.742)	1.742	0.082	5.093 (0.991; 42.181)	**Model 7**	
miR-17-5p	2.533 (1.127; 4.672)	2.877	0.004	12.592 (3.087; 106.945)	1	0.7105
(Intercept)	1.136 (−0.568; 3.188)	1.218	0.223	3.115 (0.566; 24.245)	**Model 8**	
miR-101-3p	0.985 (0.33; 1.887)	2.533	0.011	2.679 (1.391; 6.598)	0.8	0.7895

## Data Availability

Data are contained within the article.

## Supplementary Materials

The supporting information can be downloaded at: https://www.mdpi.com/article/10.3390/ijms25020871/s1.
